# Replicable association between human cytomegalovirus infection and reduced white matter fractional anisotropy in major depressive disorder

**DOI:** 10.1038/s41386-021-00971-1

**Published:** 2021-01-26

**Authors:** Haixia Zheng, Maurizio Bergamino, Bart N. Ford, Rayus Kuplicki, Fang-Cheng Yeh, Jerzy Bodurka, Kaiping Burrows, Tulsa 1000 Investigators, Robin Aupperle, Jerzy Bodurka, Justin Feinstein, Sahib S. Khalsa, Martin P. Paulus, Jonathan Savitz, Teresa A. Victor, Peter W. Hunt, T. Kent Teague, Michael R. Irwin, Robert H. Yolken, Martin P. Paulus, Jonathan Savitz

**Affiliations:** 1 Laureate Institute for Brain Research, Tulsa, OK USA; 2 Division of Neuroimaging Research, Barrow Neurological Institute, Phoenix, AZ USA; 3 Department of Neurological Surgery, School of Medicine, University of Pittsburgh, Pittsburgh, PA USA; 4 Stephenson School of Biomedical Engineering, University of Oklahoma, Norman, OK USA; 5 Department of Medicine, School of Medicine, The University of California, San Francisco, San Francisco, CA USA; 6 Department of Surgery, University of Oklahoma School of Community Medicine, Tulsa, OK USA; 7 Department of Psychiatry, University of Oklahoma School of Community Medicine, Tulsa, OK USA; 8 Department of Biochemistry and Microbiology, Oklahoma State University Center for Health Sciences, Tulsa, OK USA; 9 Cousins Center for Psychoneuroimmunology at UCLA, Los Angeles, CA USA; 10 Semel Institute for Neuroscience at UCLA, Los Angeles, CA USA; 11 David Geffen School of Medicine, Los Angeles, CA USA; 12 Stanley Division of Developmental Neurovirology, Johns Hopkins School of Medicine, Baltimore, MD USA; 13 Oxley College of Health Sciences, The University of Tulsa, Tulsa, OK USA

**Keywords:** Risk factors, Neuroimmunology

## Abstract

Major depressive disorder (MDD) is associated with reductions in white matter microstructural integrity as measured by fractional anisotropy (FA), an index derived from diffusion tensor imaging (DTI). The neurotropic herpesvirus, human cytomegalovirus (HCMV), is a major cause of white matter pathology in immunosuppressed populations but its relationship with FA has never been tested in MDD despite the presence of inflammation and weakened antiviral immunity in a subset of depressed patients. We tested the relationship between FA and HCMV infection in two independent samples consisting of 176 individuals with MDD and 44 healthy controls (HC) (Discovery sample) and 88 participants with MDD and 48 HCs (Replication sample). Equal numbers of HCMV positive (HCMV+) and HCMV negative (HCMV−) groups within each sample were balanced on ten different clinical/demographic variables using propensity score matching. Anti-HCMV IgG antibodies were measured using a solid-phase ELISA. In the Discovery sample, significantly lower FA was observed in the right inferior fronto-occipital fasciculus (IFOF) in HCMV+ participants with MDD compared to HCMV− participants with MDD (cluster size 1316 mm^3^; *p*_FWE_ < 0.05, *d* = −0.58). This association was confirmed in the replication sample by extracting the mean FA from this exact cluster and applying the identical statistical model (*p* < 0.05, *d* = −0.45). There was no significant effect of diagnosis or interaction between diagnosis and HCMV in either sample. The effect of chronic HCMV infection on white matter integrity may—in at-risk individuals—contribute to the psychopathology of depression. These findings may provide a novel target of intervention for a subgroup of patients with MDD.

## Introduction

Inflammatory processes have been hypothesized to play a significant role in the development of major depressive disorder (MDD) [1–3]. This pattern of chronic inflammation tends to co-occur with impairments to the adaptive immune system, weakening anti-viral immunity in MDD [4–7]. Specifically, in vitro studies of immune cells from depressed and chronically-stressed individuals are indicative of a decreased proliferative response of lymphocytes to mitogens, decreased natural killer cell function, and lymphopenia [8, 9]. Gene expression studies indicate downregulated expression of genes involved in anti-viral immunity [4–6]. In vivo, experimental studies demonstrate that subjects exposed to rhinovirus or influenza are more likely to become infected and show clinical symptoms if they endorse recent stress or are lonely [10–13]. Further, depression is associated with impairment of vaccine-induced immunity to the varicella-zoster virus [14, 15] and hepatitis B virus [16], a loss of childhood vaccine-induced immunity to measles [17], and impaired control of chronic viral infections [18–20]. This study focuses on the potential sequalae of one such chronic infection in MDD—human cytomegalovirus (HCMV).

HCMV is a common herpesvirus that establishes lifelong latent infections in approximately 50% of the US population via its ability to manipulate and evade the immune system [21, 22]. HCMV persists in myeloid lineage cells but can also infect endothelial cells of the blood-brain barrier, glia, and neurons [23–26]. This neurotropism may explain why HCMV is an important cause of neurological disease in HIV patients and why HCMV infection in utero can have serious neurodevelopmental and neuropsychological sequelae, including mental retardation, cerebral palsy, and sensorineural hearing loss [27–29]. In contrast, primary HCMV infection and its periodic reactivation are usually considered benign in medically-healthy populations. However, activation of latent HCMV infection can occur in response to inflammatory challenge or stress [30–35]. Some evidence suggests that such activation contributes to inflammation-related pathology in several autoimmune disorders [36], and is a risk factor for adverse outcomes in sepsis patients [37]. Notably, these pathological effects may extend to the brain. For instance, HCMV positive (HCMV+) multiple sclerosis patients showed greater brain atrophy over time than HCMV negative (HCMV−) patients [38] and higher HCMV IgG titers (indicative of greater HCMV reactivation) during a first demyelinating event predicted greater loss of gray matter volume over time [38]. Similarly, higher lifetime HCMV antibody levels and a greater CD4^+^ response to HCMV antigen were associated with the presence of neurofibrillary tangles and a diagnosis of Alzheimer’s disease at postmortem [39].

Activation of the sympathetic nervous system, possibly induced by stress, is thought to be an effector mechanism that promotes reactivation of HCMV [30]. Higher HCMV IgG titers have been reported in medical students during exams [31, 32], in female caregivers to disabled children [35], and in astronauts directly before and after space travel [33, 34]. Given the well-established association between MDD, stress, and inflammation [1, 2], it is conceivable that HCMV may be more prone to reactivation in MDD populations. Indeed, HCMV has been associated with depression in at least 13 studies [40–53]. In our recent work, we found that HCMV infection was associated with reduced gray matter volume in participants with MDD but not healthy controls [54]. However, the link between HCMV and brain abnormalities in the context of psychiatric illness has received very little attention despite the fact that there may be potential treatment implications given the availability of anti-HCMV medications and the ongoing development of HCMV vaccines [55, 56].

The oligodendroglia responsible for white matter myelination are thought to be particularly vulnerable to the damaging effects of viral infections and inflammatory processes [3, 57, 58], potentially explaining why one of the most prominent findings in MDD at postmortem is a reduction in the number or density of oligodendrocytes [3, 59]. The fractional anisotropy (FA) value derived from diffusion tensor imaging (DTI) has been reported to be highly sensitive to axon myelination [60, 61] (although it likely also serves as a general index of neuronal integrity reflecting a combination of myelination, neural fiber compactness, axon diameter, and orientation [62]). Thus, this study investigated whether HCMV serostatus was associated with white matter microstructural integrity (FA) in the context of MDD. We hypothesized that relative to seronegative MDD participants, seropositive MDD participants would show reduced FA of major white matter tracts in the brain.

## Methods

### Participants

Two independent groups of participants were involved in the study, 303 participants in the Discovery sample, and 202 in the Replication sample. Both Discovery and Replication sample participants were aged 18–55 years and either had no personal history of psychiatric illness (healthy controls, HC) or received a DSM-V diagnosis of MDD (with or without comorbid anxiety) based on the Mini International Neuropsychiatric Inventory (MINI) [63]. Participants completed the Patient-Reported Outcomes Measurement Information System (PROMIS) [64] scales for depression and anxiety, Patient Health Questionnaire 9 (PHQ-9) [65] for depressive symptoms, Customary Drinking and Drug use Record (CDDR) structured interview for lifetime alcohol use [66], as well as the childhood trauma questionnaire (CTQ) for early life stress [67]. For the Discovery sample, data were collected between January 2015 and February 2017. Exclusion criteria included: comorbid psychiatric disorders (except for anxiety disorders), substance use disorders (except for alcohol use disorder), neurological disorders, unstable medical disorders, a history of moderate-to-severe traumatic brain injury, a positive urine drug screen, a body mass index (BMI)<17 or >38 kg/m^2^, and general MRI exclusion criteria (details in the ref. [68]). For the Replication sample, data were collected from October 2018 and March 2020. The same exclusion criteria applied to the Replication sample except that the BMI cut-off was 40 and participants with a history of autoimmune disorders (except hypothyroidism) were also excluded. Approval for both studies was obtained from the Western Institutional Review Board and written informed consent was obtained from all participants.

### Anti-CMV IgG antibodies and C-reactive protein

Plasma (Discovery sample) or serum (Replication sample) were isolated from morning blood samples following standard laboratory procedures and frozen at −80 °C. Thawed samples were tested blind to diagnosis for IgG antibodies using a solid-phase ELISA (IBL America, catalog #EI2570-9601G). A sample was considered HCMV seropositive if it had an optical density value 20% over the supplied cutoff standard, which is equivalent to approximately ten international units of antibody. Due to the non-normal distribution, the density values were quantified as plate-adjusted *z*-scores with a mean value for each plate of two and a standard deviation of one.

For the Discovery sample, serum concentrations of c-reactive protein (CRP) were analyzed with the V-PLEX Neuroinflammation Panel-1 Human Kit (Meso Scale Diagnostics) with the lowest level of quantification (LLOQ) of 0.027 mg/L and intra-assay and inter-assay coefficients of variation of 2 and 10%, respectively. For the replication sample, CRP was measured using venous whole blood with the Diazyme high sensitivity (hs) CRP point of care (POC) test kit (#DZ135B-SMA-discontinued) on the SMART 700 analyzer (Diazyme Laboratories). The measurement range was from 0.5 to 23 mg/L.

### Image acquisition

Diffusion MRI scans were acquired using two identical 3.0 T scanners (GE Discovery MR750) with brain-dedicated receive-only 32 element coil arrays optimized for parallel imaging (Nova Medical, Inc.). For the Discovery sample, the diffusion-weighted imaging (DWI) data were acquired using a single-shell acquisition with 60 diffusion encoding directions (*b* value = 1000 s/mm^2^, TR/ TE = 9000/83.6 ms, with acquisition and reconstruction matrix = 128 × 128, field of view (FOV) = 25.6 × 25.6 cm, slice thickness = 2 mm, without interslice spacing, 73 axial slices, acceleration factor *R* = 2 in the phase encoding direction) and 8 no diffusion-weighted images (*b* value = 0 s/mm^2^) acquired at beginning of the scan. The total acquisition time was 10 min and 50 s.

For the replication sample, the DWI data were acquired using a multiband sequence with acceleration factor 3 and multi-shell acquisition with 102 diffusion encoding directions (*b* values = 500, 1000, 2000, and 3000 s/mm^2^, TR/ TE = 4100/81.7 ms, with acquisition and reconstruction matrix = 140 × 140, field of view (FOV) = 24.0 × 24.0 cm, slice thickness = 1.7 mm, without interslice spacing, 80 axial) and 12 no diffusion-weighted images. Total acquisition time was 7 min and 27 s. For this sequence, a reverse phase-encoding acquisition with six no diffusion-weighted images (*b* = 0 s/mm^2^) and six diffusion-weighted images (*b* = 3000 s/mm^2^) was acquired to correct for EPI image distortions.

### Individual-level image processing

DWI data were preprocessed using the FMRIB Software Library tool (FSL, version 6.0, ﻿https://fsl.fmrib.ox.ac.uk/fsl). Similar preprocessing steps were performed on both samples. The FSL “eddy” tool was used to estimate and correct eddy current-induced distortions and gross participant movement [69]. The quality of the dataset was assessed using the eddy QC tools [70]. Slices with signal loss caused by participant movement coinciding with the diffusion encoding were detected and replaced by predictions made by a Gaussian Process [71]. The quality control criteria were set as average absolute volume to volume head motion of <3 mm or total outliers <5%. Skull stripping was performed for each participant using FSL-Bet [72]. Tensor fitting and FA calculations were performed using the FSL-dtifit tool at each voxel in native space [73]. The Advanced Normalization Tools (ANTs) with asymmetric image normalization method co-registration algorithm was employed to normalize the FA maps to the FMRIB58_FA_1mm template [74]. The normalized images were visually inspected for alignment and then smoothed with a Gaussian kernel of 2 mm sigma (approximately 4.7 mm full width at half maximum) to increase the signal-to-noise ratio. A threshold of FA greater than 0.2 was used to construct a binary white mater mask across all participants. To minimize the partial volume effect, the final white matter mask consisted only of voxels that were nonzero in 100% of the sample. The Replication sample, only, was collected with reversed phase-encode blips which allowed for distortion correction. Therefore, the distortion correction using FSL-topup tool [75] was applied to the replication sample but not to the discovery sample.

### Covariates and propensity score matching

To minimize potential selection bias on HCMV status, a propensity score matching approach was used in both discovery and replication samples. First, following the principles of confounder selection [76], ten variables that could theoretically influence the likelihood of HCMV infection, or cause white matter structure change, or both, were selected as potential confounders, i.e., age, sex, BMI, education, early-life stress (total CTQ score), psychotropic medication status, the severity of current symptoms of depression and anxiety, number of episodes (obtained from MINI interview), and the lifetime alcohol use (obtained from CDDR interview). Second, a small number of missing data points (<3%) for these ten variables (Supplementary Table S8) were imputed by using the *k* nearest neighbor algorithm with *k* = 10 (R, DMwR package). Third, a multivariable logistic regression model was used to estimate the individual propensity score, which was defined as the likelihood of being HCMV+ conditioned on the given set of covariates. Then a 1-to-1 matching (without caliper) was carried out to match HCMV+ participants with HCMV− participants based on the nearest propensity score. This propensity matching process was implemented in MDD and HC groups separately. For the MDD group, the HCMV+ and HCMV− subgroups were matched on all the ten covariates mentioned above. For the HC group, the HCMV+ and HCMV− subgroups were matched on the same variables except for medication status. Thus, the matching process ensured that groups had similar baseline characteristics with respect to the given set of covariates, in theory, only differing on HCMV status. Additionally, for the Discovery sample, HC participants were matched with MDD participants on age, sex, BMI, and education using a ratio of 1:4. However, because the effect of depression on FA was not the main research question, in order to include as many participants as possible in Replication sample, we did not perform this between diagnostic group matching for the Replication sample.

### Group-level statistical analysis

To test the effects of HCMV serostatus on white matter microstructure, whole-brain voxel-wise analyses were performed using 3dMVM, an AFNI-based multivariate modeling program [77]. Although we applied propensity score matching to adjust for selection bias, it was not possible to achieve an ideal balance of covariates (i.e., all the covariates’ standardized mean differences between HCMV+ and HCMV− groups of less than 0.1) [78] without pruning too many observations given our sample size. Therefore, three common covariates used in DTI studies (i.e., age, sex, and BMI) were added in the multivariate regression model as covariates to further control for any potential imbalance that remained between the propensity-matched HCMV+ and HCMV− groups. A discovery/replication approach was used to confirm the robustness of any findings. For the Discovery sample, a multiple-comparison correction was performed to determine significant clusters using Monte-Carlo simulations through AFNI’s 3dClustSim and 3dFWHMx program which estimates spatial autocorrelation function parameters to determine the critical cluster size threshold of *p* < 0.005 with an overall family-wise error (FWE) rate of *α* < 0.05 [79, 80]. Next, we generated a region of interest (ROI) binary mask which was determined by the clusters showing significant differences (FWE-corrected) in the Discovery sample. Subsequently, the mean FA value in the same cluster was extracted from the Replication sample by using the mask generated from the Discovery sample. The same statistical model was applied to test whether the ROI mean FA from the Replication sample was also significantly associated with HCMV status. The threshold for the confirmation test was set at *p* < 0.05. Exploratory whole-brain voxel-wise analyses for the Replication sample are also reported for completeness using a voxel-level threshold of *p* < 0.05, uncorrected. A population-averaged tractography atlas (*N* = 842) and DSI Studio ﻿(http://dsi-studio.labsolver.org/) was used to identify and visualize the major white matter tracts passing through significant clusters [81].

The relationship between the mean FA from the ROI and the anti-HCMV IgG level was examined within MDD HCMV+ subgroups in both samples. CRP concentration was log-transformed and correlation analyses between ROI mean FA value and CRP concentration were performed within the MDD HCMV+ subgroups in both samples. The association between the mean FA from the ROI and depressive symptom severity (measured by each of the nine PHQ-9 items and the total PHQ-9 score) was tested in MDD groups by using a linear regression model with age, sex, and BMI as covariates. A two-sample *t*-test was performed to exam the symptom difference between HCMV− and HCMV+ subgroups in participants with MDD.

Sensitivity analyses were implemented to test the robustness of the associations. That is, we ran two additional regression models; the first not adjusting for any of the potential confounders after the matching process, and the second adjusting for all ten covariates described above as well as head motion in the scanner after the matching process. Additionally, we calculated *E*-values [82] to evaluate the robustness of the results to potential unmeasured confounding.

## Results

### Study population and covariate balance

**Fig. 1:**
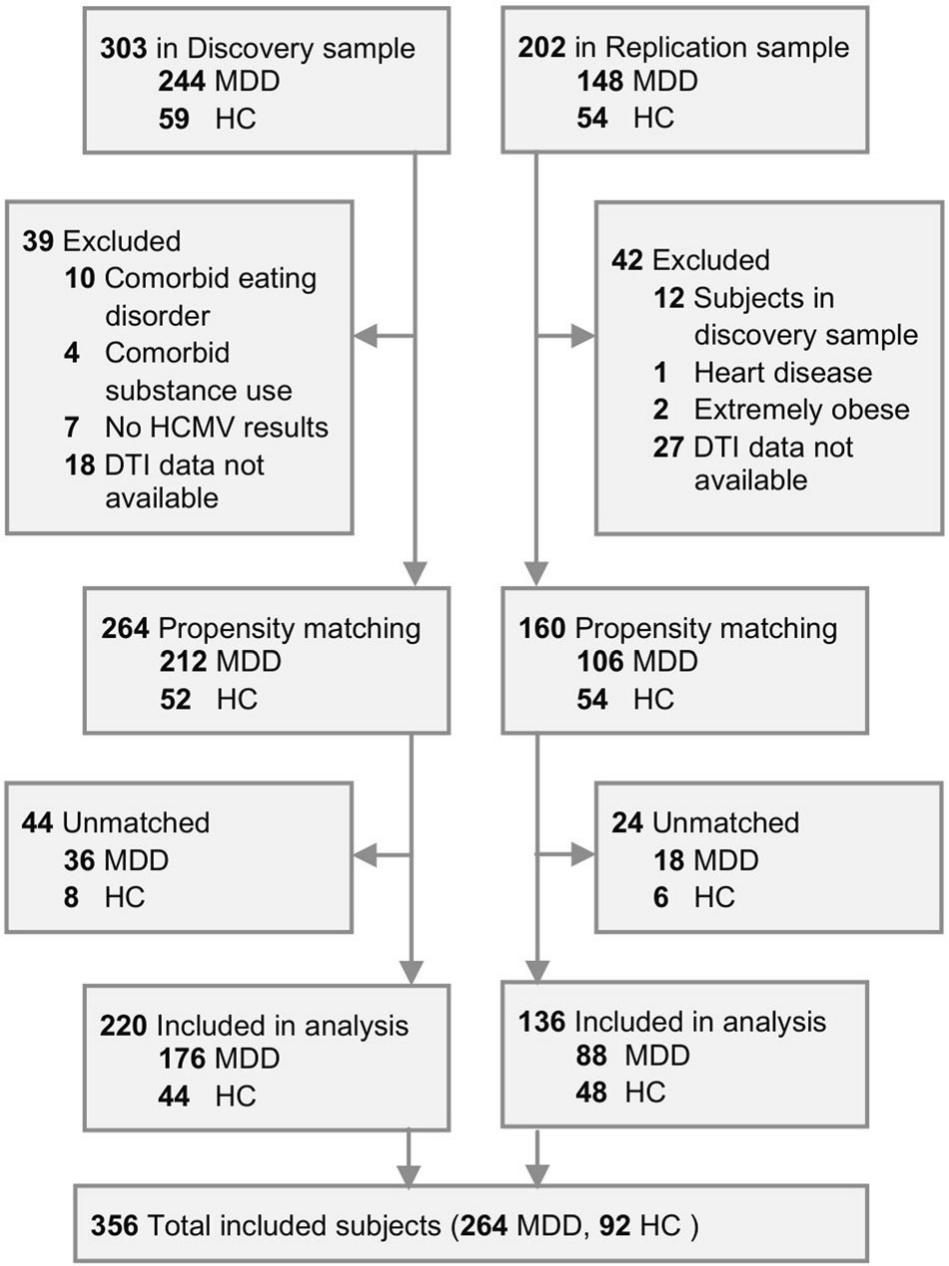
Flow diagram of selection of participants. Figure shows the detailed information for final subjects’ inclusion.

Out of a total of 303 participants in the Discovery sample, we excluded 83 participants, and out of 202 participants in the Replication sample, we excluded 66 participants, leaving a total of 356 participants included in the group-level statistical analyses (Fig. 1). Demographic characteristics before propensity matching are summarized in Supplementary Table S1. After applying propensity matching, there were no statistically significant group differences in any of the measured covariates between HCMV+ and HCMV− subgroups in both sets of MDD and HC samples (Table 1). Demographic differences between HC and MDD groups are summarized in Supplementary Table S2. Detailed medication and comorbidity characteristics of the participants with MDD are summarized in Supplementary Table S3.

**Table 1:** Demographic characteristics of study participants after applying propensity matching.

	HC				MDD			
Discovery sample	HCMV−	HCMV+	*P*^a^	SMD^b^	HCMV−	HCMV+	*P*	SMD
*n*	22	22			88	88		
Age (mean (SD))	33.50 (12.27)	33.09 (10.43)	0.91	0.04	33.07 (10.18)	34.92 (11.14)	0.25	0.17
Sex (Male (%))	10 (45.5)	11 (50.0)	1.00	0.09	32 (36.4)	27 (30.7)	0.52	0.12
BMI (mean (SD))	27.95 (5.60)	30.20 (5.19)	0.18	0.42	29.13 (5.30)	28.58 (5.15)	0.48	0.11
Education (mean (SD))^c^	7.05 (1.40)	6.36 (1.89)	0.18	0.41	6.64 (1.46)	6.64 (1.58)	0.99	0.00
Depression severity (mean (SD))^d^	45.53 (5.83)	42.82 (7.25)	0.18	0.41	61.32 (7.04)	61.93 (7.33)	0.57	0.09
Anxiety severity (mean (SD))^e^	46.98 (7.73)	43.66 (7.38)	0.15	0.44	61.90 (7.03)	62.74 (6.34)	0.41	0.13
Medicated (%)^f^	–	–	–	–	56 (63.6)	58 (65.9)	0.88	0.05
CTQ (mean (SD))^g^	32.14 (6.98)	35.73 (12.10)	0.24	0.36	45.77 (16.43)	47.61 (20.17)	0.51	0.10
Number of episodes (mean (SD))^h^	0.00 (0.00)	0.00 (0.00)	NA	0.00	3.87 (3.24)	3.88 (3.18)	0.98	0.01
Alcohol use (mean (SD))^i^	3.93 (2.40)	4.21 (2.36)	0.70	0.12	5.01 (2.21)	4.98 (2.79)	0.95	0.01
HCMV IgG level (mean (SD))^j^	0.99 (0.17)	2.56 (0.60)	<0.001	3.53	0.98 (0.17)	2.70 (0.57)	<0.001	4.11
Log CRP (mean (SD))^k^	−0.18 (1.20)	0.46 (1.60)	0.14	0.45	0.51 (1.39)	0.55 (1.49)	0.84	0.03
Head motion (SD)^l^	0.63 (0.23)	0.78 (0.51)	0.21	0.38	0.78 (0.48)	0.76 (0.35)	0.67	0.06
*Replication sample*								
*n*	24	24			44	44		
Age (mean (SD))	26.09 (6.17)	28.47 (8.88)	0.29	0.31	30.94 (11.17)	33.70 (16.83)	0.37	0.19
Sex (Male (%))	3 (12.5)	3 (12.5)	1.00	<0.001	10 (22.7)	9 (20.5)	1.00	0.06
BMI (mean (SD))	23.65 (4.95)	25.90 (5.02)	0.13	0.45	26.86 (4.84)	28.88 (5.74)	0.08	0.38
Education (mean (SD))	6.75 (1.33)	6.92 (1.35)	0.67	0.13	6.57 (1.70)	6.23 (1.65)	0.34	0.20
Depression severity (mean (SD))	43.58 (5.27)	43.65 (6.50)	0.97	0.01	62.65 (6.51)	62.41 (6.87)	0.87	0.04
Anxiety severity (mean (SD))	46.80 (5.94)	45.74 (8.65)	0.62	0.14	63.07 (6.48)	63.35 (5.67)	0.83	0.05
Medicated (%)	–	–	–	–	13 (29.5)	10 (22.7)	0.63	0.16
CTQ (mean (SD))	31.00 (5.42)	34.21 (11.68)	0.23	0.35	47.09 (16.19)	51.80 (19.98)	0.23	0.26
Number of episodes (mean (SD))	0.00 (0.00)	0.00 (0.00)	NA	0.00	3.91 (3.43)	4.37 (3.46)	0.53	0.13
Alcohol use (mean (SD))	4.37 (2.05)	3.38 (2.62)	0.15	0.42	4.52 (2.35)	4.17 (2.31)	0.49	0.15
HCMV IgG level (mean (SD))	1.24 (0.21)	3.27 (0.67)	<0.001	4.10	1.26 (0.22)	2.97 (0.59)	<0.001	3.84
Log CRP (mean (SD))	0.19 (0.84)	0.28 (0.75)	0.73	0.11	0.91 (1.07)	0.72 (0.95)	0.40	0.19
Head motion (SD)	0.96 (0.37)	0.96 (0.45)	0.99	0.00	0.96 (0.27)	0.97 (0.36)	0.93	0.02

*MDD* major depressive disorder, *HC* healthy control, *HCMV* human cytomegalovirus, *HCMV−* human cytomegalovirus seronegative, HCMV+ human cytomegalovirus seropositive, *SMD* standardized mean difference, *BMI* body mass index, *CTQ* childhood trauma questionnaire, *CRP* C-reactive protein

^a^Calculated using *X*^2^ test for categorical variables and two-tailed *t*-test for continuous variables.

^b^The standardized mean differences less than 0.1 reveals a negligible imbalance.

^c^Measured by ordered categories. Full categories see Supplementary Table S7.

^d^PROMIS depression *T*-score was used.

^e^PROMIS anxiety *T*-score was used.

^f^Medicated defined as patients with MDD taking psychotropic medication.

^g^Childhood trauma questionnaire total score was used.

^h^Measured by MINI interview. Participants with over ten episodes were treated as having had ten episodes.

^i^Log-transformed lifetime alcohol usage were used. Data obtained from CDDR interview.

^j^HCMV IgG level *z*-score was used.

^k^CRP concentration (log transformed).

^l^Average absolute volume to volume head motion (mm) in the scanner was used.

### Effect of HCMV

**Fig. 2:**
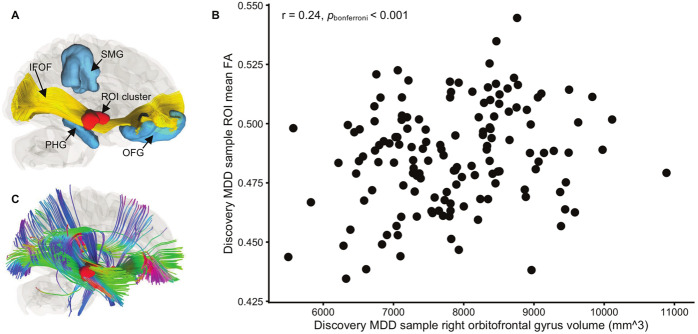
Illustration of the white matter fiber tract associated with HCMV infection. **A** The red region of interest (ROI) was determined by the clusters showing significantly lower FA (pFWE < 0.05) in HCMV+ participants with MDD compared to HCMV− participants with MDD in the Discovery sample. A population-averaged tractography atlas (*N* = 842) was used to identify the right inferior fronto-occipital fasciculus (IFOF), which passes 100% through the ROI cluster. Interestingly, three right hemisphere gray matter regions, the orbitofrontal gyrus (OFG), the parahippocampal gyrus (PHG) and the supramarginal gyrus (SMG) showed reduced volume in HCMV+ participants with MDD compared to HCMV− participants with MDD in the Discovery sample (previously published in the ref. [54]). **B** There was a significant positive association (*r* = 0.24, *p*_bonferroni_ < 0.001) between the mean FA from the ROI and the orbitofrontal gyrus volume in Discovery MDD sample (gray matter volume data from the ref. [54]). This association was also significant after regressing out age, sex, BMI, and total intracranial volume (standardized beta coefficient = 0.20, [95% CI, 0.08–0.31], *P* = 0.001). No significant associations were found with the PHG and the SMG. **C** Illustration of putative connections between the ROI and other brain regions. A deterministic fiber-tracking was performed using a published population-average template (HCP-842 template). Tractography was conducted using the DSI Studio with default parameter setting, and 50,000 seeding regions (starting points) were placed across the whole brain. The same cluster identified from the Discovery sample was used as the region of interest (ROI), which was used to “filter in” the tracks that passed through this region. A total of 829 tracts that passing through ROI was identified and shown in **C**. Although IFOF is the major track that passes through the ROI cluster, whole brain tractography suggest that the ROI may connect with the temporal lobe, parietal lobe, and prefrontal cortex via other pathways such as the inferior longitudinal fasciculus and the superior longitudinal fasciculus. Thus, it is important to note that the effect of HCMV may not be localized in IFOF.

**Fig. 3:**
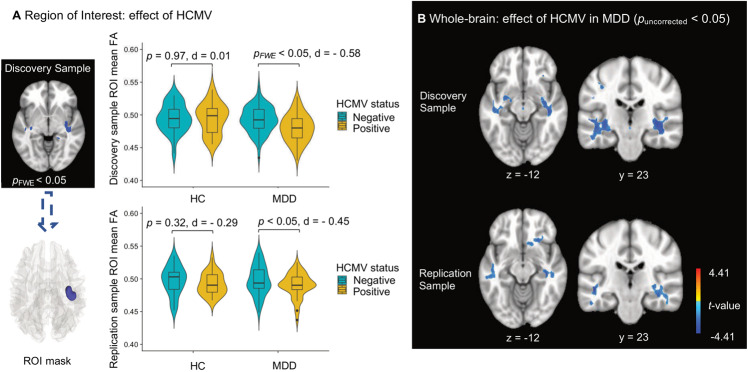
HCMV seropositive participants with MDD exhibited significantly lower white matter fractional anisotropy in both samples. **A** In the Discovery sample, significantly lower FA (*p*_FWE_ < 0.05) was observed in the right inferior fronto-occipital fasciculus in HCMV+ participants with MDD compared to HCMV− participants with MDD. Significantly lower mean FA in HCMV+ participants with MDD compared to HCMV− participants with MDD at the same cluster was confirmed in the Replication sample. Cohen’s *d* was calculated after regressing out age, sex, and BMI. **B** Exploratory whole-brain voxel-wise analyses using a voxel level threshold of *p*_uncorrected_ < 0.05 revealed the HCMV effect in MDD was bilateral in both samples.

We hypothesized that the effects of HCMV on WM microstructure would be most salient in the context of depression. We therefore tested for HCMV effects within the MDD and HC groups, separately. In the Discovery sample, significantly lower FA was observed in the right inferior fronto-occipital fasciculus (IFOF) in HCMV+ participants with MDD compared to HCMV− participants with MDD (Figs. 2 and 3). The HCMV+ and HCMV− HC groups did not differ significantly from each other. The significant cluster was selected as a region of interest (ROI) to test in the Replication sample. Lower mean FA in HCMV+ participants with MDD compared to HCMV- participants with MDD at the same cluster was confirmed in the Replication sample (Fig. 3A and Table 2). Exploratory whole-brain voxel-wise analyses at a voxel level threshold of *p* < 0.05 uncorrected, revealed lower FA in the IFOF in HCMV+ participants with MDD compared to HCMV− participants with MDD in both the left and right hemispheres in the Discovery sample. Similar, bilaterally significant results were observed in the Replication sample participants with MDD (Fig. 3B, Table 2, and Supplementary Fig. S1). Although not statistically significant, lower FA in the bilateral IFOF was also found in HCMV+ HC participants compared to HCMV− HC participants in the Discovery sample (voxel level *p*_uncorrected_ < 0.05) but not in the Replication sample (Supplementary Fig. S1).

**Table 2:** Brain regions showing significant differences in white matter FA between HCMV+ participants with MDD and HCMV− participants with MDD.

	Region	Sample	*t-*value^a^	Voxel^b^ (mm^3^)	MNI coordinates^c^		
					*X*	*Y*	*Z*
ROI analysis (*P*_FWE_ < 0.05)	R. IFOF	Discovery	−3.75	1316	−36	+13	−4
		Replication	−3.13	1316	−34	+20	−1
Whole-brain analysis (*P*_uncorrected_ < 0.05)	R. IFOF	Discovery	−3.75	3913	−36	+13	−4
		Replication	−3.41	4421	−36	+20	−14
	L. IFOF	Discovery	−3.64	3545	+51	+26	+2
		Replication	−4.41	2274	+48	+22	−6

*HCMV* human cytomegalovirus, *HCMV−* human cytomegalovirus seronegative, *HCMV+* human cytomegalovirus seropositive, *ROI* region of interest, *FWE* family-wise error rate, *R.IFOF* right inferior fronto-occipital fasciculus, *L.IFOF* left inferior fronto-occipital fasciculus, *MNI* Montreal Neurological Institute.

^a^Bi-sided cluster peak *t*-value is shown; A negative value indicates that HCMV+ participants have lower FA than HCMV− participants.

^b^Clustering method was faces or edges touch.

^c^Cluster peak MNI coordinates are shown. The *X*, *Y*, *Z* dimensions refer to left (+) to right (−), posterior (+) to anterior (−), and inferior (+) to superior (−).

In additional exploratory analyses we found that there was no significant main effect of HCMV when MDD and HC groups were combined together. Further, there was no significant main effect of diagnosis nor interaction effect of diagnosis by HCMV status in either the Discovery or the Replication samples.

Sensitivity analyses with two additional models (no covariates and eleven covariates, respectively) yielded results consistent with those reported above (Supplementary Table S4) supporting the robustness of the findings. Sensitivity analyses for unmeasured confounding suggested that the observed effect of HCMV on FA value in the right IFOF in both MDD samples was robust against unmeasured confounding. The *E*-value estimated for the right IFOF in the Discovery sample was 2.78, indicating that in order to fully explain away the observed effect of HCMV there would need to be an unmeasured confounder that increased the likelihood of being HCMV+ and reduced FA of the right IFOF by at least 2.78-fold each. Similarly, the *E*-value estimated for the effect of HCMV on the right IFOF in the Replication sample was 2.38. (Supplementary Fig. S2).

### Correlations between HCMV level, CRP, and FA

Correlation analyses were performed in the HCMV+ MDD samples. There were no significant correlations between the HCMV IgG antibody level or CRP and FA in either the Discovery or Replication samples (Supplementary Fig. S3).

### Associations between FA, HCMV serostatus, and specific depressive symptoms

Lower FA was associated with more sleep problems (standardized beta coefficient (SBC) = −0.16, [95% CI, −0.31 to −0.01], *p*_uncorrected_ < 0.05) and concentration problems (SBC = −0.15, [95% CI, −0.29 to 0.00], *p*_uncorrected_ < 0.05) in participants with MDD in the Discovery sample, but not in the Replication sample (Supplementary Table S5). The results indicated that a 1 standard deviation decrease of FA was associated with a 0.16 standard deviation increase in sleep problems and 0.15 standard deviation increase in concentration problems in participants with MDD. There were no significant differences in depressive symptoms between the HCMV+ and the HCMV− MDD groups in either the Discovery or the Replication samples (Supplementary Table S6).

## Discussion

In this study we tested the hypothesis that HCMV would be associated with reduced white matter structural integrity in individuals with MDD. The principal finding was a bilateral reduction in FA of the IFOF in two independent groups of HCMV+ versus HCMV− adults with MDD but no corresponding significant HCMV effect in HCs. The IFOF is a large white matter tract that connects the occipital lobe to the inferior frontal lobe (particularly the orbitofrontal cortex) via the insula and the posterolateral temporal lobe [83]. The IFOF is involved in semantic language processing [84] but perhaps more relevant to psychiatry, connects the “salience network” to the “executive network”, and therefore plays a role in integrating emotional and cognitive stimuli to facilitate goal-oriented behavior [83, 85]. Reduced FA of the IFOF has been widely reported in MDD populations [86–96] but appears to be a non-specific finding, also being reported in bipolar disorder (BD) [89, 97, 98], outpatients with subsyndromal affective and psychotic symptoms [99], schizophrenia [100], Parkinson’s disease with psychosis [101], and individuals with a history of childhood maltreatment [102]. Indeed, a meta-analysis of five different “emotional disorders” (MDD, BD, social anxiety disorder, obsessive-compulsive disorder, and post-traumatic stress disorder) reported reductions in FA in left IFOF (as well as other regions) compared to controls [103].

Nevertheless, we note that although the IFOF is the major track that passes through the ROI cluster, whole brain tractography using an averaged template suggests that other white matter fibers such as inferior longitudinal fasciculus may also pass through the ROI. Second, analysis with a more liberal statistical threshold reveals that reductions in FA may not limited to the IFOF but include the inferior longitudinal fasciculus, superior longitudinal fasciculus, and corticospinal tract (Supplementary Fig. S1). Thus, with larger sample sizes it is conceivable that a more widespread pattern of reductions in FA would be apparent. Indeed, in a previous study, we reported reduced gray matter volume (GMV) of the right orbitofrontal cortex (OFC), parahippocampal gyrus (PHG), and supramarginal gyrus (SMG) in Discovery sample participants with MDD who were HCMV+ vs. MDD participants who were HCMV− [54]. There was a significant positive correlation between the mean FA of the ROI cluster in the IFOF and GMV of the right OFC (but not PHG or SMG) in the MDD participants of the Discovery sample (Fig. 2). These data raise the possibility that HCMV may alter the structure of a neural circuit involving the OFC. The OFC is involved in reward processing, decision making, and the regulation of negative affect [104, 105] and has been reported to be reduced in volume or thickness in large consortia studies and meta-analyses of MDD populations [106–108].

A steeper decline in OFC volumes during adolescence has been associated with anhedonia [109] and smaller OFC volumes and reduced FA in several tracts including the IFOF were recently shown to be associated with higher polygenic risk scores for anhedonia [110]. However, here we did not observe a significant relationship between mean FA in the IFOF ROI and anhedonia as measured by the first item of the PHQ-9. Rather, lower FA of the IFOF was associated with greater sleep and concentration problems in the MDD participants of the Discovery sample (Table S5) but these relationships were not significant in the Replication sample and should therefore be treated with caution. Moreover, exploratory analyses to determine if specific clusters of depressive symptoms were associated with HCMV infection yielded non-significant results (Table S6). Thus, at least in terms of the psychometric instruments administered in this study, HCMV+ and HCMV− participants with MDD do not show clear differences in depressive symptomatology.

The mechanism underlying the link between HCMV infection and reduced FA is unclear. Because HCMV is neurotrophic [23–26], viral reactivation could, in theory, damage the brain directly or elicit a microglia-mediated antiviral immune response that has detrimental effects on brain tissue. Multifocal lesions of the deep white matter are commonly detected with MRI in children with congenital HCMV infection [111–113] while T2-weighted periventricular hyperintensities are characteristic of HIV patients with HCMV encephalitis [114, 115]. Clearly these lesions are not specific to the IFOF, although the only DTI study of which we are aware did report reduced FA in white matter tracts of the occipital lobe in neonates with a postnatally-acquired HCMV infection [116]. Another mechanism through which HCMV infection may lead to microstructural changes in white matter is via systemic inflammation including the long-term accumulation of cytotoxic CD28^−^ T-cells [36]. Indeed, reduced white matter integrity (including FA of the IFOF) of both MDD [93] and BD [117] participants has been previously associated with increased serum pro-inflammatory cytokine concentrations. Negative correlations between FA and circulating inflammatory mediators have also been reported in schizophrenia [118], Alzheimer’s disease [119], and healthy adults [120, 121]. In this regard, the absence of a significant correlation between CRP and FA in the current study was unexpected. It is conceivable that CRP does not adequately capture this brain–immune relationship, since the aforementioned studies reported associations between FA and inflammatory cytokines rather than CRP. Specific markers of viral infection such as CXCL10/IP-10 or macrophage activation such as sCD14 may also be more sensitive to HCMV reactivation than CRP [122]. Another possibility is that that the single time point at which CRP was measured did not always overlap with viral shedding since the two studies were not designed to enroll participants with an active HCMV infection.

We did not find a significant association between HCMV IgG level and FA. Leboyer and colleagues reported an inverse correlation between HCMV IgG level and right hippocampal volumes in patients with schizophrenia and BD [123], and in the Discovery sample we recently reported a similar relationship between HCMV infection and GMV of the right orbitofrontal gyrus and right PHG [54], which are located adjacent to the IFOF (Fig. 2). However, IgG antibody titer only provides an approximate measure of HCMV shedding since IgG antibodies have a half-life of <30 days and are also influenced by host factors. Thus, the signal-to-noise ratio of these correlational analyses are likely to be low.

Because of the cross-sectional design we cannot conclude that HCMV is the cause of the reduction in FA since an unknown causal factor may co-occur with HCMV infection. Nevertheless, we attempted to mitigate confounding bias through two strategies. First, we matched HCMV+ and HCMV− groups on the basis of ten potential confounders. Second, the statistical models further adjusted for all the measured confounders in the sensitivity analyses. Thus, any imbalances that remained after the matching process were controlled for by the statistical analysis. In particular, we attempted to match the groups on childhood trauma which we previously found to be more prevalent in HCMV+ individuals with MDD [124] as well as education level, a surrogate marker for childhood socioeconomic status [125]. Both childhood trauma and socioeconomic status have previously been associated with reductions in white matter integrity [102, 126]. However, it is important to acknowledge that we could not directly control for childhood socioeconomic status. We computed *E*-values to estimate the magnitude of the effect an unmeasured confounding factor(s) would need to have in order to explain away the HCMV effect. The estimated E-value was much greater than the magnitude of the effect of the largest known confounder, i.e., age, indicating that the HCMV effect observed in current study is unlikely to be easily eliminated by further inclusion of confounders.

Notwithstanding the caveats in drawing causal conclusions about HCMV, our results raise the possibility that periodic reactivation of the virus may negatively affect brain structure, thus contributing to the emergence or maintenance of depressive symptoms. Conceivably, FDA-approved medications for the treatment of HCMV such as valganciclovir or letermovir [55, 127–129] may have therapeutic or prophylactic effects in a subgroup of patients. A clinical trial in HIV patients demonstrated that 8 weeks of anti-HCMV therapy with valganciclovir reduced CD8+ cell activation and CRP, sCD14, and TNFR2 concentrations by ∼1 quartile, an effect that persisted for at least 4 weeks after valganciclovir discontinuation [130]. Testing whether an immune-modulating effect of anti-HCMV medication could modify brain structure and/or reduce depressive symptoms in the context of MDD is indicated.

Several limitations deserve mention. Although we did not observe a significant main effect of diagnosis or interaction effect on FA, these results should be interpreted with caution. The current study was not designed to investigate the effect of depression on FA. That is, the propensity matching focused on HCMV status, not diagnosis, and further, the small sample size of the HC groups limited the statistical power available to detect a main effect of diagnosis and an interaction effect. Second, the cross-sectional design did not allow us to differentiate between possible acute and cumulative effects of HCMV on brain structure. Third, it is conceivable that other viral infections that co-occur with HCMV may have accounted for the reductions in FA. Nevertheless, HCMV is more strongly linked with congenital brain abnormalities than other herpesviruses and recurrent HCMV reactivation disrupts the balance of the immune system to a greater extent than other herpesviruses [131]. Finally, while FA is usually considered to be a general measure of microstructural integrity of the white matter, reductions in FA are multifactorial [60, 132, 133]. Thus, the biological correlates of the changes in FA associated with CMV infection are unclear and are not necessarily reflective of a neuropathological process.

In sum, after careful balancing of HCMV+ and HCMV− groups for ten baseline demographic and clinical variables to minimize confounding bias, we found evidence for an HCMV-associated reduction in FA of the IFOF in two independent MDD samples. While causal conclusions cannot be drawn from these cross-sectional analyses, the results offer a new perspective on the origin of structural brain abnormalities in a HCMV+ subgroup of patients with MDD. They also potentially open-up new avenues of treatment given the availability of anti-HCMV medications.

## Funding and disclosure

This work was supported by The William K. Warren Foundation, the National Institute of Mental Health (R21MH11387), and the National Institute of General Medical Sciences (P20GM121312). The funding sources had no role in the design and conduct of the study: collection, management, analysis, and interpretation of the data; preparation, review, or approval of the manuscript; and decision to submit the manuscript for publication.

Dr. Hunt receives support from Merck (donated drug) for a clinical trial of letermovir. Dr. Paulus is an advisor to Spring Care, Inc., a behavioral health startup, he has received royalties for an article about methamphetamine in UpToDate. All other authors, including members of Tulsa 1000 Investigators, have no disclosures to report.

## Supplementary information

Supplemental material

## Data Availability

The full preprocessing script, statistical analysis code, and unthresholded statistical imaging maps are available from the corresponding author on reasonable request.
