# Supplementary material for: Replicable association between human cytomegalovirus infection and reduced white matter fractional anisotropy in major depressive disorder
Source: Neuropsychopharmacology. 2021 Jan 26;46(5):928–38. doi: 10.1038/s41386-021-00971-1 (PMC8115597; doi:10.1038/s41386-021-00971-1)
Supplement: Supplementary file 1 — Supplemental material [file 41386_2021_971_MOESM1_ESM.doc]

**Supplementary Contents**

Table of Contents

[Supplementary Table S1. Demographic differences between HCMV status (before applying propensity matching) 2](#__RefHeading___Toc59543198)

[Supplementary Table S2. Demographic differences between HC and MDD participants (before and after applying propensity matching) 4](#__RefHeading___Toc59543199)

[Supplementary Table S3. Medication and comorbidity characteristics of the participants with MDD 6](#__RefHeading___Toc59543200)

[Supplementary Table S4. Sensitivity analysis to the choice of measured covariates 7](#__RefHeading___Toc59543201)

[Supplementary Table S5. Associations between the mean FA from the ROI and depressive symptoms. 8](#__RefHeading___Toc59543202)

[Supplementary Table S6. Differences in specific depressive symptoms (PHQ-9 items) between HCMV- and HCMV+ participants with MDD. 9](#__RefHeading___Toc59543203)

[Supplementary Table S7. Education measurement 10](#__RefHeading___Toc59543204)

[Supplementary Table S8. Number of missing data for covariates 10](#__RefHeading___Toc59543205)

[Supplementary Figure S1. HCMV effect in MDD and HC participants. 11](#__RefHeading___Toc59543206)

[Supplementary Figure S2. Sensitivity analysis to the unmeasured confounder 12](#__RefHeading___Toc59543207)

[Supplementary Figure S3. Results of correlation analyses 14](#__RefHeading___Toc59543208)

### Supplementary Table S1. Demographic differences between HCMV status (before applying propensity matching)

|  | **HC** | | | | | **MDD** | | | |
| --- | --- | --- | --- | --- | --- | --- | --- | --- | --- |
| **Discovery Sample** | **HCMV-** | **HCMV+** | ***P^a^*** | **SMD^b^** | **HCMV-** | | **HCMV+** | ***P*** | **SMD** |
| n | 30 | 22 |  |  | 91 | | 121 |  |  |
| Age (mean (SD)) | 32.85 (12.02) | 33.09 (10.43) | 0.94 | 0.02 | 33.37 (10.30) | | 37.76 (11.39) | <0.01 | 0.41 |
| Sex (Male (%)) | 13 (43.3) | 11 (50.0) | 0.85 | 0.13 | 32 (35.2) | | 29 (24.0) | 0.10 | 0.25 |
| BMI (mean (SD)) | 26.60 (5.55) | 30.20 (5.19) | 0.02 | 0.67 | 28.90 (5.37) | | 28.89 (5.47) | 0.99 | 0.00 |
| Education (mean (SD))^c^ | 7.03 (1.43) | 6.36 (1.89) | 0.15 | 0.40 | 6.62 (1.47) | | 6.14 (1.78) | 0.04 | 0.29 |
| Depression severity (mean (SD))^d^ | 44.82 (5.86) | 42.82 (7.25) | 0.28 | 0.30 | 61.02 (7.13) | | 61.84 (7.63) | 0.43 | 0.11 |
| Anxiety severity (mean (SD))^e^ | 47.17 (7.58) | 43.66 (7.38) | 0.10 | 0.47 | 61.85 (6.98) | | 63.24 (6.33) | 0.14 | 0.21 |
| Medicated (%)^f^ | -- | -- | -- | -- | 58 (63.7) | | 84 (69.4) | 0.47 | 0.12 |
| CTQ (mean (SD))^g^ | 30.80 (6.41) | 35.73 (12.10) | 0.06 | 0.51 | 45.43 (16.38) | | 52.67 (21.98) | 0.01 | 0.37 |
| Number of episodes (mean (SD))^h^ | 0.00 (0.00) | 0.00 (0.00) | NA | 0.00 | 3.95 (3.35) | | 4.30 (3.56) | 0.49 | 0.10 |
| Alcohol use (mean (SD))^i^ | 4.05 (2.67) | 4.20 (2.42) | 0.83 | 0.06 | 5.06 (2.27) | | 5.19 (2.71) | 0.72 | 0.05 |
| HCMV IgG level (mean (SD))^j^ | 0.97 (0.17) | 2.56 (0.60) | <0.001 | 3.57 | 0.98 (0.17) | | 2.80 (0.60) | <0.001 | 4.12 |
| Log CRP (mean (SD))^k^ | -0.31 (1.16) | 0.46 (1.60) | 0.05 | 0.55 | 0.47 (1.40) | | 0.66 (1.52) | 0.35 | 0.13 |
| Head motion (SD)^l^ | 0.61 (0.21) | 0.78 (0.51) | 0.10 | 0.44 | 0.79 (0.49) | | 0.82 (0.49) | 0.71 | 0.05 |
| **Replication Sample** |  |  |  |  |  | |  |  |  |
| n | 30 | 24 |  |  | 62 | | 44 |  |  |
| Age (mean (SD)) | 25.16 (6.02) | 28.47 (8.88) | 0.11 | 0.44 | 30.16 (11.19) | | 31.63 (10.54) | 0.50 | 0.14 |
| Sex (Male (%)) | 6 (20.0) | 3 (12.5) | 0.71 | 0.20 | 13 (21.0) | | 9 (20.5) | 1.00 | 0.01 |
| BMI (mean (SD)) | 24.02 (4.73) | 25.90 (5.02) | 0.16 | 0.39 | 25.54 (4.78) | | 28.88 (5.74) | <0.01 | 0.63 |
| Education (mean (SD)) | 6.10 (1.81) | 6.92 (1.35) | 0.07 | 0.51 | 6.71 (1.54) | | 6.23 (1.65) | 0.13 | 0.30 |
| Depression severity (mean (SD)) | 43.59 (5.23) | 43.65 (6.50) | 0.97 | 0.01 | 63.51 (6.72) | | 62.41 (6.87) | 0.41 | 0.16 |
| Anxiety severity (mean (SD)) | 46.54 (6.26) | 45.74 (8.65) | 0.70 | 0.11 | 63.14 (6.35) | | 63.35 (5.67) | 0.86 | 0.04 |
| Medicated (%) | -- | -- | -- | -- | 22 (35.4) | | 10 (22.7) | 0.23 | 0.28 |
| CTQ (mean (SD)) | 30.07 (5.25) | 34.21 (11.68) | 0.09 | 0.46 | 44.76 (15.00) | | 51.80 (19.98) | 0.04 | 0.40 |
| Number of episodes (mean (SD)) | 0.00 (0.00) | 0.00 (0.00) | NA | 0.00 | 3.93 (3.51) | | 4.42 (3.49) | 0.49 | 0.14 |
| Alcohol use (mean (SD)) | 3.67 (2.48) | 3.38 (2.62) | 0.68 | 0.11 | 4.42 (2.33) | | 4.20 (2.33) | 0.64 | 0.10 |
| HCMV IgG level (mean (SD)) | 1.23 (0.21) | 3.27 (0.67) | <0.001 | 4.11 | 1.26 (0.22) | | 2.97 (0.59) | <0.001 | 3.88 |
| Log CRP (mean (SD)) | 0.10 (0.81) | 0.28 (0.75) | 0.44 | 0.23 | 0.72 (1.02) | | 0.72 (0.95) | 0.99 | 0.00 |
| Head motion (SD) | 0.95 (0.34) | 0.96 (0.45) | 0.93 | 0.02 | 1.00 (0.33) | | 0.97 (0.36) | 0.65 | 0.09 |

Abbreviations: MDD = major depressive disorder; HC = healthy control; HCMV = human cytomegalovirus; HCMV- = human cytomegalovirus seronegative; HCMV+ = human cytomegalovirus seropositive; SMD = standardized mean difference; BMI = body mass index; CTQ = childhood trauma questionnaire. CRP = C-reactive protein.

^a^ Calculated using X^2^ test for categorical variables and 2-tailed t-test for continuous variables. ^b^ The standardized mean differences less than 0.1 reveals a negligible imbalance. ^c^ Measured by ordered categories. Full categories see supplement Supplementary Table S7. ^d^ PROMIS depression T score was used. ^e^ PROMIS anxiety T score was used. ^f^ Medicated defined as taking psychotropic medication. ^g^ Childhood trauma questionnaire total score was used. ^h^ Measured by MINI interview. Participants with over 10 episodes were treated as having had 10 episodes. ^i^ Log-transformed lifetime alcohol usage were used. Data obtained from CDDR interview. ^j^ HCMV IgG level z score was used. ^k^ CRP concentration log transferred. ^l^ Average absolute volume to volume head motion (mm) in the scanner was used.

### Supplementary Table S2. Demographic differences between HC and MDD participants (before and after applying propensity matching)

|  | **Before HCMV status Matching** | | | | | **After HCMV status Matching** | | | |
| --- | --- | --- | --- | --- | --- | --- | --- | --- | --- |
| **Discovery Sample** | **HC** | **MDD** | ***P^a^*** | **SMD^b^** | **HC** | | **MDD** | ***P*** | **SMD** |
| n | 52 | 212 |  |  | 44 | | 176 |  |  |
| Age (mean (SD)) | 32.95 (11.27) | 35.88 (11.12) | 0.09 | 0.26 | 33.30 (11.26) | | 34.00 (10.68) | 0.70 | 0.06 |
| Sex (Male (%)) | 24 (46.2) | 61 (28.8) | 0.03 | 0.37 | 21 (47.7) | | 59 (33.5) | 0.12 | 0.29 |
| BMI (mean (SD)) | 28.12 (5.64) | 28.89 (5.41) | 0.36 | 0.14 | 29.07 (5.45) | | 28.86 (5.22) | 0.81 | 0.04 |
| Education (mean (SD))^c^ | 6.75 (1.66) | 6.35 (1.67) | 0.12 | 0.24 | 6.70 (1.68) | | 6.64 (1.52) | 0.80 | 0.04 |
| Depression severity (mean (SD))^d^ | 43.98 (6.49) | 61.49 (7.41) | <0.001 | 2.51 | 44.17 (6.65) | | 61.62 (7.17) | <0.001 | 2.52 |
| Anxiety severity (mean (SD))^e^ | 45.69 (7.63) | 62.64 (6.64) | <0.001 | 2.37 | 45.32 (7.65) | | 62.32 (6.69) | <0.001 | 2.37 |
| Medicated (%)^f^ | -- | 142 (67.0) | -- | -- | -- | | 114 (64.8) | -- | -- |
| CTQ (mean (SD))^g^ | 32.88 (9.47) | 49.56 (20.06) | <0.001 | 1.06 | 33.93 (9.93) | | 46.69 (18.37) | <0.001 | 0.86 |
| Number of episodes (mean (SD))^h^ | -- | 4.15 (3.47) | -- | -- | -- | | 3.88 (3.20) | -- | -- |
| Alcohol use (mean (SD))^i^ | 4.11 (2.54) | 5.13 (2.53) | 0.01 | 0.40 | 4.07 (2.36) | | 4.99 (2.51) | 0.03 | 0.38 |
| HCMV IgG level (mean (SD))^j^ | 1.64 (0.89) | 2.02 (1.02) | 0.02 | 0.39 | 1.77 (0.91) | | 1.84 (0.96) | 0.67 | 0.07 |
| Log CRP (mean (SD))^k^ | 0.02 (1.40) | 0.58 (1.47) | 0.01 | 0.39 | 0.14 (1.43) | | 0.53 (1.44) | 0.11 | 0.27 |
| Head motion (SD)^l^ | 0.68 (0.38) | 0.81 (0.49) | 0.08 | 0.30 | 0.70 (0.40) | | 0.77 (0.42) | 0.36 | 0.16 |
| **Replication Sample** |  |  |  |  |  | |  |  |  |
| n | 54 | 106 |  |  | 48 | | 88 |  |  |
| Age (mean (SD)) | 26.63 (7.54) | 30.77 (10.90) | 0.01 | 0.44 | 27.28 (7.66) | | 32.32 (14.27) | 0.03 | 0.44 |
| Sex (Male (%)) | 9 (16.7) | 22 (20.8) | 0.68 | 0.11 | 6 (12.5) | | 19 (21.6) | 0.28 | 0.24 |
| BMI (mean (SD)) | 24.85 (4.90) | 26.93 (5.43) | 0.02 | 0.40 | 24.77 (5.06) | | 27.87 (5.37) | 0.00 | 0.59 |
| Education (mean (SD)) | 6.46 (1.66) | 6.51 (1.60) | 0.86 | 0.03 | 6.83 (1.33) | | 6.40 (1.68) | 0.12 | 0.29 |
| Depression severity (mean (SD)) | 43.62 (5.77) | 63.05 (6.77) | <0.001 | 3.09 | 43.62 (5.86) | | 62.53 (6.66) | <0.001 | 3.02 |
| Anxiety severity (mean (SD)) | 46.18 (7.35) | 63.22 (6.05) | <0.001 | 2.53 | 46.27 (7.36) | | 63.21 (6.05) | <0.001 | 2.51 |
| Medicated (%) | -- | 32 (30.2) | -- | -- | -- | | 23 (26.1) | -- | -- |
| CTQ (mean (SD)) | 31.91 (8.87) | 47.68 (17.50) | <0.001 | 1.14 | 32.60 (9.16) | | 49.44 (18.23) | <0.001 | 1.17 |
| Number of episodes (mean (SD)) | -- | 4.14 (3.49) | -- | -- | -- | | 4.14 (3.43) | -- | -- |
| Alcohol use (mean (SD)) | 3.54 (2.52) | 4.33 (2.32) | 0.05 | 0.33 | 3.87 (2.38) | | 4.35 (2.32) | 0.26 | 0.20 |
| HCMV IgG level (mean (SD)) | 2.14 (1.12) | 1.97 (0.94) | 0.31 | 0.17 | 2.25 (1.14) | | 2.12 (0.96) | 0.46 | 0.13 |
| Log CRP (mean (SD)) | 0.17 (0.79) | 0.72 (0.99) | 0.001 | 0.61 | 0.23(0.79) | | 0.81 (1.01) | 0.001 | 0.64 |
| Head motion (SD) | 0.95 (0.39) | 0.99 (0.34) | 0.55 | 0.10 | 0.96 (0.41) | | 0.97 (0.31) | 0.87 | 0.03 |

Abbreviations: MDD = major depressive disorder; HC = healthy control; HCMV = human cytomegalovirus; HCMV- = human cytomegalovirus seronegative; HCMV+ = human cytomegalovirus seropositive; SMD = standardized mean difference; BMI = body mass index; CTQ = childhood trauma questionnaire. CRP = C-reactive protein.

^a^ Calculated using X^2^ test for categorical variables and 2-tailed t-test for continuous variables. ^b^ The standardized mean differences less than 0.1 reveals a negligible imbalance. ^c^ Measured by ordered categories. Full categories see supplement Supplementary Table S7. ^d^ PROMIS depression T score was used. ^e^ PROMIS anxiety T score was used. ^f^ Medicated defined as taking psychotropic medication. ^g^ Childhood trauma questionnaire total score was used. ^h^ Measured by MINI interview. Participants with over 10 episodes were treated as having had 10 episodes. ^i^ Log-transformed lifetime alcohol usage were used. Data obtained from CDDR interview. ^j^ HCMV IgG level z score was used. ^k^ CRP concentration log transferred. ^l^ Average absolute volume to volume head motion (mm) in the scanner was used.

### Supplementary Table S3. Medication and comorbidity characteristics of the participants with MDD

|  | **Discovery Sample** | | | | | **Replication Sample** | | | | |
| --- | --- | --- | --- | --- | --- | --- | --- | --- | --- | --- |
|  | HCMV- |  | HCMV+ |  |  | HCMV- |  | HCMV+ |  |  |
|  | Med | Un-med | Med | Un-med | *p* | Med | Un-med | Med | Un-med | *p* |
| n | 56 | 32 | 58 | 30 |  | 13 | 31 | 10 | 34 |  |
| Medicated (%) | 56 (63.6) |  | 58 (65.9) |  | 0.88 | 13 (29.5) |  | 10 (22.7) |  | 0.63 |
| Type of medication (%) |  |  |  |  |  |  |  |  |  |  |
| SSRI | 21 (37.5) | 0 | 19 (32.8) | 0 |  | 8 (61.5) | 0 | 5 (50.0) | 0 |  |
| SNRI | 8 (14.3) | 0 | 7 (12.1) | 0 |  | 1 (7.7) | 0 | 1 (10.0) |  |  |
| NDRI | 2 (3.6) | 0 | 2 (3.4) | 0 |  | 4 (30.8) |  | 1 (10.0) |  |  |
| Benzodiazepine | 7 (12.5) | 0 | 7 (12.1) | 0 |  |  |  |  |  |  |
| Multiple classes | 14 (25.0) | 0 | 17 (29.3) | 0 |  |  |  |  |  |  |
| Serotonin partial agonist | 0 (0.0) | 0 | 1 (1.7) | 0 |  |  |  |  |  |  |
| TCA | 1 (1.8) | 0 | 0 (0.0) | 0 |  |  |  |  |  |  |
| Glutamate channel blocker | 0 (0.0) | 0 | 1 (1.7) | 0 |  |  |  |  |  |  |
| Muscle Relaxant | 3 (5.4) | 0 | 3 (5.2) | 0 |  |  |  |  |  |  |
| Other | 0 (0.0) | 0 | 1 (1.7) | 0 |  | 0 |  | 3 (30.0) | 0 |  |
| NSAID | 8 (14.3) | 6 (18.8) | 12 (20.7) | 6 (20.0) | 0.58 | NA | NA | NA | NA | NA |
| Comorbidity (%) |  |  |  |  | 0.21 |  |  |  |  | 0.31 |
| Dep | 17 (30.4) | 7 (21.9) | 22 (37.9) | 11 (36.7) |  | 6 (46.2) | 11 (35.5) | 2 (20.0) | 5 (14.7) |  |
| Dep+Alc | 1 (1.8) | 2 (6.2) | 0 (0.0) | 1 (3.3) |  | 0 (0.0) | 1 (3.2) | 1 (10.0) | 0 (0.0) |  |
| Dep+GAD | 19 (33.9) | 11 (34.4) | 11 (19.0) | 9 (30.0) |  | 6 (46.2) | 14 (45.2) | 3 (30.0) | 15 (44.1) |  |
| Dep+PD | 7 (12.5) | 0 (0.0) | 9 (15.5) | 2 (6.7) |  | 0 (0.0) | 1 (3.2) | 0 (0.0) | 1 (2.9) |  |
| Dep+PTSD | 6 (10.7) | 4 (12.5) | 8 (13.8) | 5 (16.7) |  | 1 (7.7) | 3 (9.7) | 2 (20.0) | 7 (20.6) |  |
| Dep+SP | 6 (10.7) | 8 (25.0) | 8 (13.8) | 2 (6.7) |  | 0 (0.0) | 1 (3.2) | 2 (20.0) | 6 (17.6) |  |

Abbreviations: HCMV- = human cytomegalovirus seronegative; HCMV+ = human cytomegalovirus seropositive; Med = Medicated; Un-med = Un-medicated; SSRI = selective serotonin reuptake inhibitor; SNRI = selective norepinephrine reuptake inhibitor; NDRI = norepinephrine-dopamine reuptake inhibitor; TCA = tricyclic antidepressant; NSAID = nonsteroidal anti-inflammatory drug; Dep = depression (no comorbidity); Dep+Alc = depression with alcohol dependence disorder; Dep+GAD = depression with generalized anxiety disorder; Dep+PD = depression with panic disorder; Dep+PTSD = depression with post-traumatic stress disorder; Dep+SP = depression with social phobia. Note: data obtained from the MINI clinical interview. *p* value was calculated by using X^2^ test.

### Supplementary Table S4. Sensitivity analysis to the choice of measured covariates

Two additional models yielded similar results to the model reported in the manuscript.

| Sensitivity analyses to measured covariates | Approach | Region | Sample | *t-*value^a^ | Voxel^b^  (mm^3^) | MNI coordinates^c^ | | |
| --- | --- | --- | --- | --- | --- | --- | --- | --- |
|  |  |  |  |  |  | X | Y | Z |
| Model 1,  With no covariates | ROI-based analysis^d^  (*P*_FWE_ < 0. 05) | R. IFOF | Discovery | -3.96 | 1316 | -35 | +13 | -3 |
|  |  |  | Replication | -2.85 | 1316 | -34 | +20 | -1 |
|  | Whole-brain voxel-wise analysis  (*P*_uncorrected_ < 0.05) | R. IFOF | Discovery | -3.96 | 4773 | -35 | +13 | -3 |
|  |  |  | Replication | -3.13 | 2193 | -37 | +21 | -14 |
|  |  | L. IFOF | Discovery | -3.88 | 4750 | +50 | +27 | +2 |
|  |  |  | Replication | -4.25 | 1992 | +48 | +22 | -6 |
| Model 2,  With full covariates^e^ | ROI-based analysis  (*P*_FWE_ < 0. 05) | R. IFOF | Discovery | -3.63 | 1316 | -36 | +13 | -4 |
|  |  |  | Replication | -2.80 | 1316 | -34 | +20 | -1 |
|  | Whole-brain voxel-wise analysis  (*P*_uncorrected_ < 0.05) | R. IFOF | Discovery | -3.63 | 3981 | -36 | +13 | -4 |
|  |  |  | Replication | -3.22 | 2440 | -36 | +20 | -14 |
|  |  | L. IFOF | Discovery | -3.75 | 3078 | +51 | +26 | +2 |
|  |  |  | Replication | -3.07 | 882 | +52 | +28 | -17 |

Abbreviations: HCMV = human cytomegalovirus; HCMV-, human cytomegalovirus seronegative; HCMV+, human cytomegalovirus seropositive; ROI, region of interest; FWE, family-wise error rate; R.IFOF, right inferior fronto-occipital fasciculus; L.IFOF, left inferior fronto-occipital fasciculus. MNI: Montreal Neurological Institute.

^a^ Bi-sided cluster peak *t*-value is shown; A negative value indicates that HCMV+ participants had lower FA than HCMV- participants. ^b^ clustering method was faces or edges touch; ^c^ cluster peak MNI coordinates are shown. The X, Y, Z dimensions refer to left (+) to right (-), posterior (+) to anterior (-), and inferior (+) to superior (-). ^d^ The same ROI mask used in the main analysis was used here for ROI-based analysis in both samples. Mean FA value from the ROI was used as the outcome. ^e^ Covariates including age, sex, BMI, education, depression severity, anxiety severity, medication status, childhood trauma score, number of episodes, lifetime alcohol use, and head motion.

### Supplementary Table S5. Associations between the mean FA from the ROI and depressive symptoms.

The region of interest (ROI) binary mask was determined by the clusters showing significant differences (FWE-corrected) in the Discovery sample. The mean FA value in the same cluster was extracted from the Discovery sample and the Replication sample. A linear regression model controlling for age, sex, and BMI was used to estimate the association between mean FA from the ROI and PHQ-9 depressive symptoms. Each of the nine items from the PHQ-9 was analyzed separately. Higher FA was associated with less sleep problems (standardized beta coefficient (SBC)=-0.16, [95%CI, -0.31 to -0.01], *p*_uncorrected_<0.05) and less concentration problems (SBC=-0.15, [95%CI, -0.29 to 0.00], *p*_uncorrected_<0.05) in the Discovery sample, but not in the Replication sample. Detailed effect sizes and the 95% CI for each symptom are listed in the table below.

|  | **Discovery Sample** | | | **Replication Sample** | | |
| --- | --- | --- | --- | --- | --- | --- |
| Symptoms | SBC^a^ | 95% CI | *p_uncorrected_* | SBC | 95% CI | *p_uncorrected_* |
| Anhedonia^b^ | -0.08 | -0.23 ~ 0.06 | 0.26 | -0.01 | -0.21 ~ 0.20 | 0.94 |
| Depressed mood^b^ | -0.08 | -0.23 ~ 0.07 | 0.31 | -0.10 | -0.31 ~ 0.11 | 0.36 |
| Sleep problems^b^ | -0.16 | -0.31 ~ -0.01 | 0.04* | 0.08 | -0.14 ~ 0.29 | 0.48 |
| Tiredness^b^ | -0.10 | -0.25 ~ 0.05 | 0.18 | -0.08 | -0.30 ~ 0.13 | 0.45 |
| Changes in appetite^b^ | 0.00 | -0.15 ~ 0.15 | 0.97 | -0.03 | -0.25 ~ 0.19 | 0.80 |
| Feelings of inadequacy^b^ | 0.03 | -0.12 ~ 0.18 | 0.70 | 0.00 | -0.22 ~ 0.22 | 0.99 |
| Concentration problems^b^ | -0.15 | -0.29 ~ 0.00 | 0.05* | 0.02 | -0.20 ~ 0.24 | 0.87 |
| Psychomotor changes^b^ | -0.03 | -0.18 ~ 0.12 | 0.66 | -0.09 | -0.31 ~ 0.13 | 0.42 |
| Suicidality^b^ | -0.05 | -0.20 ~ 0.10 | 0.49 | -0.17 | -0.38 ~ 0.04 | 0.12 |
| PHQ-9 total score | -0.11 | -0.26 ~ 0.04 | 0.14 | -0.06 | -0.28 ~ 0.16 | 0.59 |
| Anxiety^c^ | -0.03 | -0.18 ~0.12 | 0.69 | 0.08 | -0.13 ~ 0.03 | 0.45 |
| Number of episodes^d^ | -0.02 | -0.17 ~ 0.13 | 0.76 | -0.06 | -0.27 ~ 0.15 | 0.60 |

Abbreviation: ROI, region of interest; SBC, Standardized beta coefficient; PHQ, patient health questionnaire. 95%CI, 95% confidence interval. ^a^ A linear regression model controlling for age, sex, and BMI was used to estimate the SBC and 95%CI. ^b^ Measured by PHQ-9 item. ^c^ PROMIS anxiety T score. ^d^ From MINI interview. Participants who reported over 10 episodes were treated as having had 10 episodes.

### Supplementary Table S6. Differences in specific depressive symptoms (PHQ-9 items) between HCMV- and HCMV+ participants with MDD.

|  | **Discovery Sample** | | | **Replication Sample** | | |
| --- | --- | --- | --- | --- | --- | --- |
|  | **HCMV-** | **HCMV+** | ***p*** | **HCMV-** | **HCMV+** | ***p*** |
| n | 88 | 88 |  | 44 | 44 |  |
| Anhedonia (mean (SD)) | 1.60 (0.86) | 1.56 (0.83) | 0.72 | 1.34 (0.71) | 1.18 (0.62) | 0.27 |
| Depressed mood (mean (SD)) | 1.56 (0.84) | 1.51 (0.80) | 0.71 | 1.30 (0.90) | 1.41 (0.62) | 0.49 |
| Sleep problems (mean (SD)) | 1.99 (0.94) | 2.15 (0.93) | 0.26 | 1.93 (1.04) | 1.59 (1.09) | 0.14 |
| Tiredness (mean (SD)) | 2.09 (0.83) | 2.11 (0.90) | 0.86 | 2.07 (0.87) | 1.84 (0.89) | 0.23 |
| Changes in appetite (mean (SD)) | 1.49 (1.08) | 1.52 (1.02) | 0.83 | 1.07 (0.85) | 1.27 (1.04) | 0.32 |
| Feelings of inadequacy (mean (SD)) | 1.86 (0.98) | 1.72 (1.02) | 0.32 | 1.27 (0.79) | 1.45 (0.79) | 0.28 |
| Concentration problems (mean (SD)) | 1.40 (0.98) | 1.45 (1.03) | 0.71 | 1.34 (0.89) | 1.18 (0.97) | 0.43 |
| Psychomotor changes (mean (SD)) | 0.67 (0.77) | 0.76 (0.88) | 0.47 | 0.48 (0.70) | 0.32 (0.60) | 0.26 |
| Suicidality (mean (SD)) | 0.44 (0.66) | 0.44 (0.72) | 1.00 | 0.43 (0.76) | 0.30 (0.51) | 0.33 |
| PHQ-9 total score (mean (SD)) | 13.10 (4.98) | 13.23 (4.93) | 0.87 | 11.23 (4.36) | 10.55 (4.01) | 0.45 |

Abbreviations: HCMV-, human cytomegalovirus seronegative; HCMV+, human cytomegalovirus seropositive; PHQ-9, patient health questionnaire. 2-tailed t-test was used to calculate the *p* value.

### Supplementary Table S7. Education measurement

Education was measured by 11 categories for both samples, and treated as a continuous variable:

1. No school through kindergarten
2. Grade 1-11
3. Grade 12, no diploma
4. Regular HS diploma
5. GED or equivalent
6. Some college, no degree
7. Associate's degree
8. Bachelor's degree
9. Master's degree
10. Professional degree beyond a bachelor's
11. Doctoral degree

### Supplementary Table S8. Number of missing data for covariates

|  | Age | Sex | BMI | Education | Depression severity | Anxiety severity | Medication status | CTQ | Number of episodes | Lifetime Alcohol use |
| --- | --- | --- | --- | --- | --- | --- | --- | --- | --- | --- |
| Discovery Sample | 0 | 0 | 4 | 0 | 2 | 2 | 0 | 0 | 7 | 8 |
| Replication Sample | 0 | 0 | 0 | 1 | 0 | 0 | 0 | 0 | 2 | 1 |

### Supplementary Figure S1. HCMV effect in MDD and HC participants.

Exploratory whole-brain voxel-wise analyses using a voxel level threshold of *p*_uncorrected_ < 0.05 revealed that the HCMV effect in MDD was bilateral in both samples. HCMV+ HC participants also showed reduced FA in bilateral inferior fronto-occipital fasciculus in the Discovery sample but not in the Replication sample.


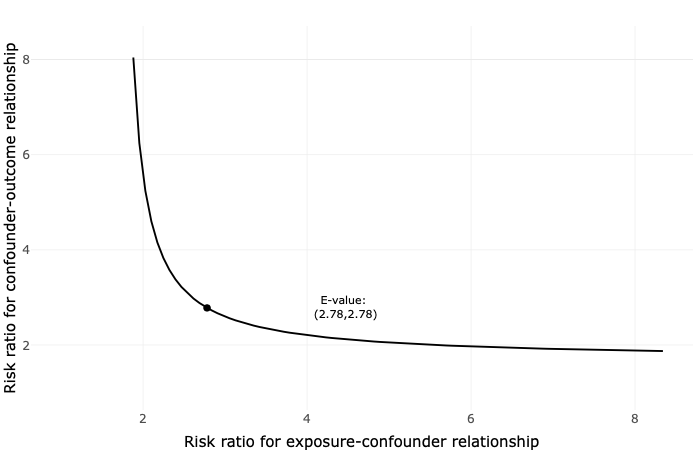


### Supplementary Figure S2. Sensitivity analysis to the unmeasured confounder

The figure shows the estimated E-value for the observed effect of HCMV in the right inferior fronto-occipital fasciculus in MDD participants of the Discovery sample. The E-value methodology estimates the joint minimum strength of association on the risk ratio scale that an unmeasured confounder must have with both treatment and outcome to fully explain away an observed effect. In the current study, E-Value was estimated based on effect size which was estimated from the linear regression model using the participants with MDD mean FA value from the significant cluster (region of interest) as the outcome, HCMV status as the exposure with the full set of measured confounders (ten variables described in main article) and head motion in scanner as covariates. The estimated E-value of 2.78 (confidence interval (CI), 1.92) for the effect of HCMV in Discovery sample indicates that in order to explain away the observed effect of HCMV in the Discovery sample (standardized beta coefficient = -0.58, standard error = 0.15), an unmeasured confounder would need to increase the probability of a participant being HCMV+ and having a lower FA value at same region by 2.79-times each, above and beyond the measured confounders. Weaker confounding would not explain away the association with HCMV. The CI of 1.93 indicates that to shift the observed upper confidence interval to include the null value, an unmeasured confounder would need to increase the probability of a participant being HCMV+ and having a lower FA value at the same region by at least 1.93-times each. Each point along the curve defines a joint relationship between the two sensitivity parameters that could potentially explain away the estimated effect. If one of the two parameters is smaller than the E-value, the other must be larger, as defined by the plotted curve. Similarly, based on the observed effect of HCMV serostatus on the right inferior fronto-occipital fasciculus in the participants with MDD in the Replication sample (standardized beta coefficient = -0.45, standard error = 0.21), the estimated E-value was 2.38 (CI, 1.23).

To provide a more intuitive interpretation of the E-value (sensitivity to unmeasured confounding), we used the Discovery sample to calculate the risk ratio of age for being HCMV+ and having a lower FA value at right IFOF (i.e. smaller than sample mean), controlling for the other nine measured confounders and head motion in scanner (using ‘logisticRR’ package). The results showed that increasing age by 1 standard deviation (11.1 years in the current sample), increases the risk of being HCMV+ 1.40 times, and increases the risk of having a lower FA value in right IFOF 1.09 times. Given that the estimated E-values (risk ratio, ranged from 2.38 to 2.78) are much greater than one of the largest known confounders (i.e., age), this indicates that the observed associations between HCMV seropositivity and decreased white matter FA value are robust to unmeasured confounding.

### Supplementary Figure S3. Results of correlation analyses

No significant correlation was observed between ROI mean FA value and CRP concentration or HCMV IgG level in either of the samples.
